# Atomic Scale Simulation on the Anti-Pressure and Friction Reduction Mechanisms of MoS_2_ Monolayer

**DOI:** 10.3390/ma11050683

**Published:** 2018-04-27

**Authors:** Yang Liu, Yuhong Liu, Tianbao Ma, Jianbin Luo

**Affiliations:** State Key Laboratory of Tribology, Tsinghua University, Beijing 100084, China; yang-liu@outlook.com (Y.L.); mtb@tsinghua.edu.cn (T.M.)

**Keywords:** MoS_2_ monolayer, indentation, rupture, scratch, friction, molecular dynamics

## Abstract

MoS_2_ nanosheets can be used as solid lubricants or additives of lubricating oils to reduce friction and resist wear. However, the atomic scale mechanism still needs to be illustrated. Herein, molecular simulations on the indentation and scratching process of MoS_2_ monolayer supported by Pt(111) surface were conducted to study the anti-pressure and friction reduction mechanisms of the MoS_2_ monolayer. Three deformation stages of Pt-supported MoS_2_ monolayer were found during the indentation process: elastic deformation, plastic deformation and finally, complete rupture. The MoS_2_ monolayer showed an excellent friction reduction effect at the first two stages, as a result of enhanced load bearing capacity and reduced deformation degree of the substrate. Unlike graphene, rupture of the Pt-supported MoS_2_ monolayer was related primarily to out-of-plane compression of the monolayer. These results provide a new insight into the relationship between the mechanical properties and lubrication properties of 2D materials.

## 1. Introduction

2D materials, such as graphene, few-layer MoS_2_ and black phosphorus, have shown great potential in many areas due to their special physical properties [1,2,3,4,5]. Their excellent mechanical performance [6,7] and high thermal conductivity [8], together with their flatness and thickness at the atomic scale [9], make some 2D materials ideal lubricants for certain areas [10], for example in micro- or nanoelectro-mechanical systems [11,12] and high density magnetic storage devices [13], where the lubricating space is limited and lubricating condition is severe [14], lubrication coatings of 2D materials are considered to be a good choice [15]. 

When used as lubricant additives or solid lubricants, graphene and few-layer MoS_2_ can improve the extreme pressure (highest load without seizure of the friction pairs) and reduce the friction of rubbing surfaces [16,17]. These excellent lubricating properties, including the anti-pressure effect and friction reduction effect, are usually attributed to the better mechanical properties of 2D materials than metal friction pairs [18]. For instance, graphene has a Young’s modulus of about 1000 GPa and a breaking strength of about 130 GPa, while the Young’s modulus of stainless steel is only 205 GPa, with a breaking strength of 0.9 Gpa [19]. Due to the high elastic modulus and breaking strength, graphene can share extra load and reduce friction before its rupture [20]. However, while the mechanical properties of the MoS_2_ monolayer are not as excellent as those of graphene, with a Young’s modulus of about 270 GPa and a breaking strength of about 30 GPa [19], the lubrication properties of few-layer MoS_2_ seem to be better. Macroscopic lubrication experiments have shown that MoS_2_ nanosheets can be more stable and effective than graphene when used as lubricant additives, with a lower friction coefficient [21] and higher extreme pressure [22]. The inconsistency of lubrication properties and mechanical properties requires detailed research on the atomic mechanism of the anti-pressure and friction reduction effect of MoS_2_ monolayer.

In this paper, in order to find out the anti-pressure mechanism of MoS_2_ monolayer, indentation process of MoS_2_ monolayer, MoS_2_-covered Pt (MoS_2_/Pt) and bare Pt substrate were studied by way of molecular dynamics simulations. After that, a contrast study on the scratch process of MoS_2_/Pt and bare Pt substrate was conducted to explore the friction reduction mechanism of the MoS_2_ monolayer. As the friction reduction effect of the monolayer rests on its ability to share extra load when covering the substrate [20], the scratch process was carried out at different indentation depths.

## 2. Simulation Details

**Figure 1:**
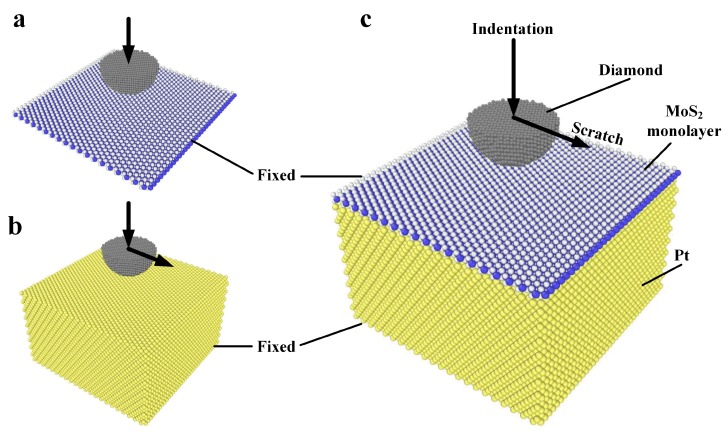
(**a**) The indentation model of freestanding MoS_2_ monolayer; (**b**) The indentation and scratch model of Pt substrate; (**c**) The indentation and scratch model of MoS_2_/Pt substrate.

In our simulation models, a rigid hemisphere tip cutout from a (111)-oriented diamond crystal is placed right above the upper surface of MoS_2_ monolayer, Pt(111) and MoS_2_/Pt substrate at an initial height (h_0_) of 10 Å, as shown in Figure 1a–c. The radius of the diamond tip was 18 Å and the size of the MoS_2_ monolayer was 104.196 × 104.478 Å^2^. Pt(111) substrate covered or not covered by a MoS_2_ monolayer has the same lateral size as the monolayer. The thickness of Pt substrate is 58.902 Å. During the indentation process, the diamond tip moves vertically to the upper surface of the substrate, while during the scratch process, the diamond tip moves laterally at different indentation depths, controlled by the vertical displacement (h) of the tip. In both indentation and scratch processes, the velocity of the tip is set to 1.0 m/s. Atoms at the edge of MoS_2_ are fixed in order to simulate large MoS_2_ layers pinned to the substrate at the edge by Van der Waals forces. Pt atoms at the bottom are also fixed to support the substrate. For MoS_2_/Pt substrate, the initial distance between the MoS_2_ monolayer and the Pt(111) surface is set to 2.8 Å, the equilibrium distance where interaction energy between these two materials is the minimum. In order to learn the structure changes caused by the mechanical effect and to avoid the affection of temperature, the initial temperature is set to 0.01 K. A Langevin thermostat [23] was used in an NVE ensemble to maintain a constant temperature of 0.01 K during the simulation of indentation and scratch. The time step in this work is 0.002 ps. The number of steps is 2 million for the indentation process of the freestanding MoS_2_ monolayer, 1.5 million for the indentation process of MoS_2_/Pt and bare Pt substrate and 1 million for the scratch process.

A modified Stilling-Webber(SW) potential parameterized by Jin-Wu Jiang was used to describe the intra-layer interaction of MoS_2_ [24]. Tersoff potential was used to describe the interaction between the carbon atoms of diamond [25]. An embedded atom method (EAM) potential was adopted to describe the interaction between Pt atoms [26]. Van der Waals forces between the diamond tip and MoS_2_ monolayer were described by C-Mo and C-S Lennard-Jones(LJ) potentials. Similarly, Van der Waals forces between the diamond tip and Pt substrate were described by a C-Pt LJ potential and Pt -Mo and Pt-S LJ potentials were used to describe the Van der Waals forces between MoS_2_ monolayer and Pt substrate in MoS_2_/Pt system. Parameters for LJ potentials mentioned above were determined by Lorentz-Berthelot mixing rules [27], with the original ε and σ parameters for C-C, Pt-Pt, Mo-Mo, S-S taken from references [28,29,30]. Parameters for all the LJ potentials used in this work are shown in Table 1. The cutoff radius for LJ potentials was 10 Å. All the simulations were carried out with the large-scale atomic/molecular massively parallel simulator (LAMMPS) [31]. 

## 3. Results and Discussions

### 3.1. Indentation Process

Indentation of freestanding 2D layers is a common method for experimentally measuring the elasticity modulus and rupture strength of 2D materials [32]. With the MoS_2_ monolayer regarded as a linear isotropic elastic material [19] and the indentation process approximated as central point loading on a clamped circular membrane [32], the relationship between indentation force and height can be deduced, as Formula (1) shows [33]:

F = σ_0_^2D^ π(h − h_0_) + E^2D^q^3^(h − h_0_)^3^/a^2^, (1)

where F is the point load at the center of the membrane, h is the vertical displacement of the tip, h − h_0_ can represent the deflection at the center point approximately, a is the radius of MoS_2_ monolayer, σ_0_^2D^ and E^2D^ are the pretension and elastic modulus of the membrane, q = 1/(1.05 − 0.15ν − 0.16ν^2^) is a dimensionless constant, ν = 0.125 is the Poisson ratio of bulk MoS_2_ [34].

**Figure 2:**
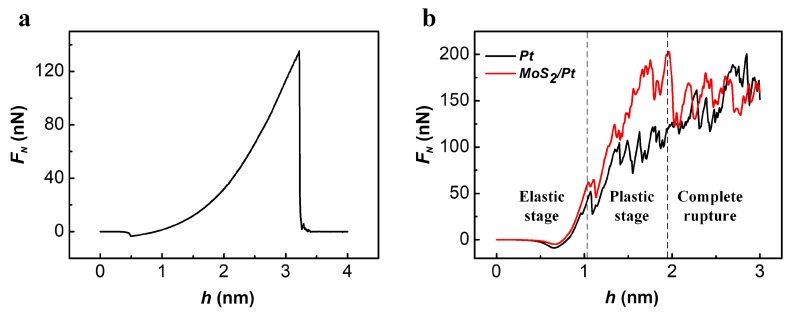
(**a**) The force-displacement curve for the indentation process of the freestanding MoS_2_ monolayer; (**b**) The force-height curve for the indentation process of bare Pt and MoS_2_/Pt substrate.

Here, in our simulation of the indentation process of freestanding MoS_2_ monolayer, the elasticity properties of MoS_2_ monolayer can be deduced by fitting the F_N_(h) curve at the elastic stage shown in Figure 2a to Formula (1). The results of E^2D^ was 199.1 N/m，which were in good agreement with the experiments [19,35]. The rupture strength was 33.8 N/m, which was very close to the results of density functional theory (DFT) calculations by Si Xiong [36]. 

As the freestanding MoS_2_ ruptured completely in a short time during the indentation process, it was difficult to distinguish the plastic deformation stage from the force height curve before rupture, as Figure 2a shows. However, the indentation process of MoS_2_/Pt substrate showed clearly the plastic deformation stage of the MoS_2_ monolayer. As shown in Figure 2b, the F_N_(h) curve can be divided into three stages: elastic deformation stage (see smooth part of F_N_(h) curve in Figure 2b, with h < 1.07 nm for MoS_2_/Pt substrate, h < 0.86 nm for bare Pt substrate); plastic deformation stage (see the sawtooth shaped steps of F_N_(h) curve in Figure 2b, with 1.07 nm < h < 1.99 nm for MoS_2_/Pt substrate, h > 0.86nm for bare Pt substrate); and finally, the complete rupture of the MoS_2_ monolayer (see the sudden drop of F_N_(h) curve at h = 1.99 nm for MoS_2_/Pt substrate in Figure 2b). At the elastic and plastic deformation stages, the existence of the MoS_2_ monolayer improved the load bearing capacity of the substrate, as F_N_ for the MoS_2_/Pt substrate is always larger than the bare Pt substrate at the same indentation depth. The two force-height curves nearly overlapped after the complete rupture of the MoS_2_ monolayer, which meant the anti-pressure effect of the MoS_2_ monolayer completely vanished. The maximum load the MoS_2_/Pt substrate can bear at the elastic deformation stage was about 62.9 nN (see Figure 2b), much smaller than 135.3 nN of the freestanding MoS_2_ monolayer with a same size (see Figure 2a), which indicated different fracture criterions. In order to learn the fracture mechanism in these two cases, the structural deformations of the MoS_2_ monolayer during the indentation process were studied.

### 3.2. Structural Deformation

**Figure 3:**
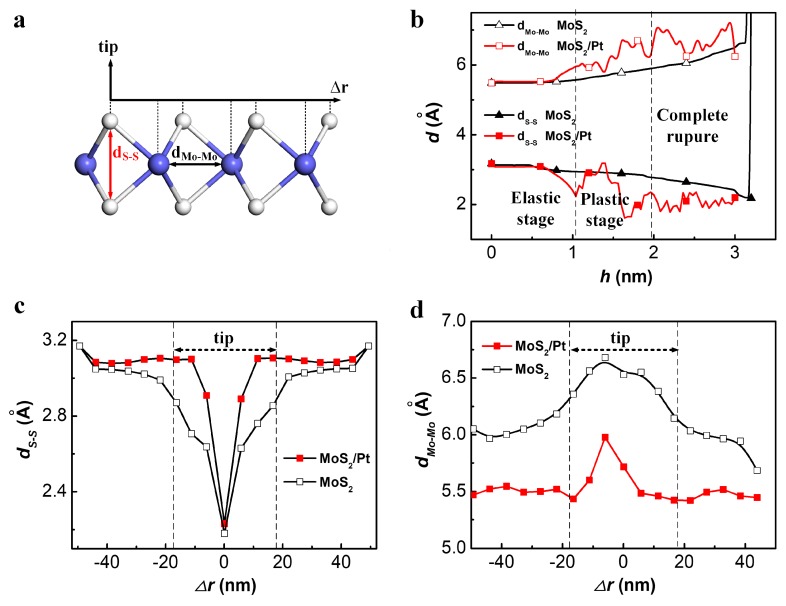
(**a**) Schematic of d_S-S_ and d_Mo-Mo_; (**b**) Evolution of d_S-S_ and d_Mo-Mo_ for atoms right under the tip during the indentation process of freestanding MoS_2_ monolayer and MoS_2_/Pt substrate; (**c**) The radial distribution of d_S-S_ for MoS_2_ monolayer and MoS_2_/Pt substrate at the end of elastic deformation stage; (**d**) The radial distribution of d_Mo-Mo_ for MoS_2_ monolayer and MoS_2_/Pt substrate at the end of elastic deformation stage.

A significant structural difference between the MoS_2_ monolayer and graphene is that the MoS_2_ monolayer consists of a molybdenum atom layer sandwiched by two sulfur layers while graphene has only one carbon atom layer. Therefore, the deformation mechanism of the MoS_2_ monolayer is different from graphene, as the out-of-plane compression of MoS_2_ monolayer exists [37]. In this paper, the main structure deformations during the indentation process were divided into two types: in-plane stretch and out-of-plane compression. The distance between two adjacent Mo atoms(d_Mo-Mo_) in the radial direction was used to represent the in-plane stretch deformation, while the distance between two opposite S atoms(d_S-S_) in the vertical direction was used to represent the out-of-plane compression deformation [38], as shown in Figure 3a.

Compared with the freestanding MoS_2_ monolayer, a higher strain rate was found in the contact region of the MoS_2_/Pt substrate, as d_S-S_ and d_Mo-Mo_ changed more rapidly for atoms right under the tip in the Pt-supported MoS_2_ monolayer than in the freestanding MoS_2_ monolayer, as shown in Figure 3b. Furthermore, the deformation was more concentrated in the contact region for MoS_2_/Pt substrate, as shown in Figure 3c,d. Similarly, with the F_N_(h) curve, both d_Mo-Mo_(h) and d_S-S_(h) were smooth at the elastic stage. The minimum d_S-S_ for bare MoS_2_ and MoS_2_/Pt substrate were very close at the end of the elasticity stage, while the maximum d_Mo-Mo_ was much smaller for MoS_2_/Pt substrate than for bare MoS_2_, as shown in Figure 3c,d. It can be speculated that the achievement of the strain limit for the out-of-plane compression is the main reason for the rupture of the MoS_2_ monolayer supported by Pt substrate, although both in-plane stretch and out-of-plane compression can bear load during the indentation process. The ultimate strain of out-of-plane compression was about 0.29 at the elastic stage for the indentation process of the MoS_2_/Pt substrate.

### 3.3. Scratch Process

**Figure 4:**
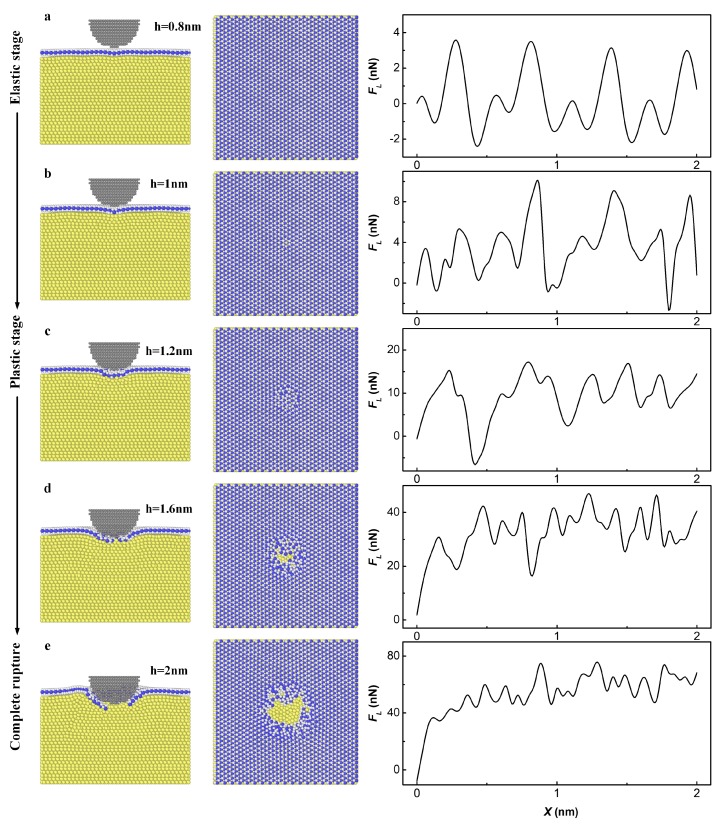
The left column shows the cutaway view of the simulation model, the middle column shows the top view of MoS_2_/Pt substrate and the right column shows variation of friction force with sliding distance at different indentation depth: (**a**,**b**) h = 0.8 and 1 nm, at elastic stage of MoS_2_ monolayer; (**c**,**d**) h = 1.2 and 1.6 nm, at plastic stage of MoS_2_ monolayer; (**e**) h = 2 nm, MoS_2_ monolayer completely ruptured.

While the results of the indentation process showed that the anti-pressure effect of the MoS_2_ monolayer is the result of structural deformation, the friction reduction effect of MoS_2_ monolayer also has a strong relationship with structural deformation. In the simulation of scratch, vertical displacement of the tip varied from 0.8 nm to more than 2 nm, thus three different deformation stages of MoS_2_ monolayer were covered. Figure 4 shows the friction changes with distance during the scratch process at different indentation depths. At the elastic stage, the friction force was small and the friction-distance curve was smooth, as shown in Figure 4a,b. Stick slip was found at a shallow indentation depth when h = 0.8 nm and disappeared when the indentation depth increased. At the plastic stage, the friction-distance curve became more and more irregular as the indentation depth increased, as shown in Figure 4c–e.

**Figure 5:**
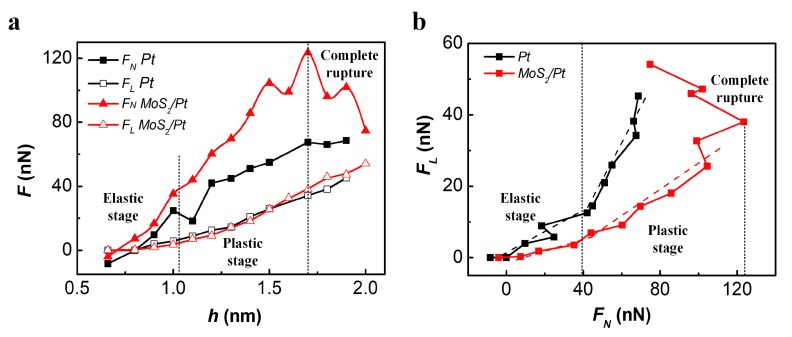
(**a**) Average load and friction varied with indentation depth during the scratch process of MoS_2_/Pt and bare Pt substrates; (**b**) The relation between average load and friction for MoS_2_/Pt and bare Pt substrates. Dashed line in (**b**) is a linear fitting of F_L_ and F_N_.

The average friction force and load at different indentation depths was calculated for the scratch process of MoS_2_/Pt and bare Pt substrates, as shown in Figure 5. Friction force increased with load at the elastic and plastic deformation stages. The friction reduction effect of MoS_2_ monolayer relied on the degree of deformation. When MoS_2_ monolayer existed, the friction coefficient was about 0.164 at the elastic stage and 0.368 at the plastic stage, which are much smaller than the 0.298 and 1.019 for the Pt substrate without MoS_2_ monolayer. The turning point for the load of MoS_2_/Pt substrate in Figure 5a,b stood for the complete rupture of the MoS_2_ monolayer. The MoS_2_ monolayer ruptured more easily in the scratch process than in the indentation process, as the maximum load MoS_2_/Pt substrate can bear before complete rupture of the MoS_2_ monolayer is about 123.5 nN for h = 1.7 nm (see Figure 5a) during the scratch process, which is much smaller than 200.5 nN for h = 1.99 nm during the indentation process (see Figure 2b). After the complete rupture of the MoS_2_ monolayer, both anti-pressure and the friction reduction effect vanished as F_N_ decreased, while F_L_ continued to increase with indentation depth for the MoS_2_/Pt substrate.

The friction for MoS_2_/Pt and bare Pt substrates were very close at the same indentation depth, while the load was much higher for MoS_2_/Pt substrate, which meant MoS_2_ monolayer can improve the load bearing capacity of the substrate without increasing friction. At the same load condition, the indentation depth for MoS_2_/Pt substrate was smaller than for bare Pt substrate and the deformation of the substrate was also smaller, which can be a result for the lower friction coefficient of MoS_2_/Pt substrate.

## 4. Conclusions

In conclusion, three deformation stages of Pt-supported MoS_2_ monolayer were observed: an elastic stage, a plastic stage and a completely ruptured stage. Unlike graphene, the MoS_2_ monolayer can share additional load through in-plane stretch and out-of-plane compression. The rupture of MoS_2_ monolayer for MoS_2_/Pt system was a result of out-of-plane compression, which is very different from graphene. When covering the Pt(111) surface, MoS_2_ monolayer can reduce deformation and increase the load bearing capacity of the substrate, which is a key reason for the friction reduction effect of MoS_2_ monolayer. The anti-pressure and friction reduction effects of MoS_2_ monolayer depend on deformation degree of the monolayer and the excellent lubrication properties will disappear after the complete rupture of the MoS_2_ monolayer. These results provide a new insight into the relationship between the mechanical properties and lubrication properties of 2D materials, which can be used to predict the application prospects of different 2D materials in tribology domain.

## Figures and Tables

**Table 1:** Parameters of Lennard-Jones (LJ) potential used in this simulation.

Pair	C-Mo	C-S	C-Pt	Pt-Mo	Pt-S
ε_ij_ (meV)	48.962	13.165	38.635	661.41	177.840
σ_ij_ (Å)	3.009	3.418	2.971	2.513	2.922
